# Supplementary material for: A Quantitative and Qualitative Program Evaluation of a Case-Area Targeted Intervention to Reduce Cholera in Eastern Democratic Republic of the Congo
Source: Int J Environ Res Public Health. 2023 Dec 23;21(1):27. doi: 10.3390/ijerph21010027 (PMC10815631; doi:10.3390/ijerph21010027)
Supplement: Supplementary file 1 [file ijerph-21-00027-s001.zip › ijerph-2693667-supplementary.pdf]

Supplemental Table S1. Participant and household demographics in rural (Katana) and urban (Bukavu) areas by CATI delivery and control areas in South Kivu, DRC.

	Katana						Bukavu					
	CATI area			Control area			CATI area			Control area		
	%	n	N	%	n	N	%	n	N	%	n	N
Households			28			30			33			41
Participants			96			89			113			101
Participants per household	3.4 ± 2.9 (1-9)			3.0 ± 3.0 (1-9)			3.4 ± 3.5 (1-13)			2.5 ± 2.4 (1-9)		
Female	57%	55	96	71%	63	89	66%	74	113	63%	64	101
Age (years)			96			89			113			101
0-2	8%	8		10%	9		12%	13		8%	8	
2-5	12%	11		11%	10		12%	13		12%	12	
5-12	21%	20		16%	14		21%	24		18%	18	
12-18	59%	57		63%	56		55%	63		62%	63	
Villages/neighborhoods		6			6			6			6	
Households per village/neighborhood	4.7 ± 2.2 (3-9)			5.0 ± 1.7 (2-6)			5.5 ± 3.4 (1-11)			6.8 ± 2.6 (5-12)		
Water Source Type§			28			30			20			27
Public tap/standpipe	7%	2		0%	0		95%	19		100%	27	
Protected dug well	43%	12		43%	13		5%	1		0%	0	
Unprotected dug well	32%	9		33%	10		0%	0		0%	0	
Tube well	11%	3		10%	3		0%	0		0%	0	
Other	7%	2		13%	4		0%	0		0%	0	
Days since CATI delivery*	12.5 ± 4.8 (5-20)			-			14.8 ± 3.2 (10-21)			-		

SD = standard deviation.

n indicates the number of participants in each category.

N indicates the total number of participants.

Households per village/neighborhood, participants per household, and days since CATI delivery were reported as Mean ± SD (min–max).

*Time since CATI delivery calculated at the household level.

CATI = case-area targeted intervention.

Supplemental Table S2. Spot checks of WASH indicators of rural (Katana) and urban (Bukavu) areas comparing CATI delivery and control areas in South Kivu, DRC.

	Katana							Bukavu						
	CATI area			Control area			p-value	CATI area			Control area			p-value
	%	n	N	%	n	N		%	n	N	%	n	N	
Households			28			30				20			27	
Villages			6			6				4			6	
Households per village														
Mean ± SD (min-max)	4.7 ± 2.2 (3-9)			5.0 ± 1.7 (2-6)				5.0 ± 2.0 (4-8)			4.5 ± 1.8 (3-8)			
The cleansing agent present in the home	25%	7	28	27%	8	30	0.885	50%	10	20	70%	19	27	0.16
Cleansing agents present within 10 steps of the latrine*	0%	0	28	10%	3	30	0.238	20%	4	20	26%	7	27	0.74
Bar soap*	0%	0	28	10%	3	30	0.238	20%	4	20	26%	7	27	0.74
Liquid soap	0%	0	28	0%	0	30	-	0%	0	20	0%	0	27	-
Soapy water	0%	0	28	0%	0	30	-	0%	0	20	0%	0	27	-
Ash	0%	0	28	0%	0	30	-	0%	0	20	0%	0	27	-
Water and cleansing agent*	4%	1	28	0%	0	29	0.491	0%	0	20	19%	5	27	0.06
Cleansing agents present within 10 steps of the cooking area*	14%	4	28	17%	5	30	1.000	20%	4	20	33%	9	27	0.35
Bar soap*	14%	4	28	13%	4	30	1.000	20%	4	20	30%	8	27	0.52
Liquid soap	0%	0	28	0%	0	30	-	0%	0	20	4%	1	27	1.00

Soapy water*	0%	0	28	3%	1	30	1.000	0%	0	20	0%	0	27	-
Ash	0%	0	28	0%	0	30	-	0%	0	20	0%	0	27	-
Water and cleansing agent*	4%	1	28	7%	2	30	1.000	20%	4	20	37%	10	27	0.33
Handwashing station present*	**25%**	**7**	**28**	**0%**	**0**	**30**	**0.004**	15%	3	20	15%	4	27	1.00
Cleansing agents within arm's reach of handwashing station*	11%	3	28	0%	0	30	0.106	5%	1	20	4%	1	27	1.00
Bar soap*	11%	3	28	0%	0	30	0.106	5%	1	20	4%	1	27	1.00
Liquid soap	0%	0	28	0%	0	30	-	0%	0	20	0%	0	27	-
Soapy water	0%	0	28	0%	0	30	-	0%	0	20	0%	0	27	-
Ash	0%	0	28	0%	0	30	-	0%	0	20	0%	0	27	-
ORS present in the home*	**46%**	**13**	**28**	**0%**	**0**	**30**	**<0.0001**	**90%**	**18**	**20**	**0%**	**0**	**27**	**<0.0001**
Chlorine tablets present in the home*	**57%**	**12**	**21**	**0%**	**0**	**11**	**0.002**	**0%**	0	15	0%	0	0	-
Stored water-free chlorine > 0.2 mg/L*	10%	2	21	4%	1	24	0.592	12%	2	17	7%	2	27	0.63
Stored water total chlorine > 0.2 mg/L*	19%	4	21	13%	3	24	0.689	12%	2	17	11%	3	27	1.00
Source water-free chlorine > 0.2 mg/L*	0%	0	27	0%	0	30	-	5%	1	18	11%	2	18	1.00
Source water total chlorine > 0.2 mg/L*	0%	0	27	10%	3	30	0.239	6%	1	18	22%	4	18	0.34

SD = standard deviation.

One spot check is conducted per household.

Cleansing agents include bar soap, liquid soap, soapy water, or ash.

*P-value calculated with Fisher's exact test.

No p-value is calculated if no observations of a specific category are recorded.

Significant findings are in bold.

CATI = case-area targeted intervention.

Supplemental Table S3. Logistic regression analysis of participants handwashing with a cleansing agent during 5-hour structured observation in rural (Katana) and urban (Bukavu) areas comparing by CATI delivery area vs. control (predictor) during household structured observation in South Kivu, DRC

	**Katana**								**Bukavu**							
	**CATI area**			**Control area**			**Logistic regression**		**CATI area**			**Control area**			**Logistic regression**	
	%	n	N	%	n	N	OR (95% CI)	p-value	%	n	N	%	n	N	OR (95% CI)	p-value
Participants handwashing with a cleansing agent at stool and food events																
All events	0%	0	50	2%	1	41	-	0.46*	2%	2	86	1%	1	75	1.74 (0.18, 17.30)	0.64
Stool events	0%	0	22	0%	0	14	-	-	3%	1	31	0%	0	39	-	0.42*
Food events	0%	0	46	3%	1	40	-	0.46*	1%	1	80	2%	1	67	0.85 (0.06, 11.97)	0.90
Participants handwashing with only water at stool and food events																
All events	0%	0	1	2%	1	41	-	0.46*	7%	6	86	3%	2	75	2.71 (0.54, 13.65)	0.23
Stool events	0%	0	22	0%	0	14	-	-	0%	0	31	0%	0	39	-	-
Food events	0%	0	46	3%	1	40	-	0.46*	8%	6	80	3%	2	67	2.64 (0.52, 13.42)	0.24

OR = odds ratio.

CI = confidence interval.

All the events include both stool and food events.

Handwashing is defined as washing both hands with a cleansing agent during structured observation.

Cleansing agents include bar soap, liquid soap, soapy water, and ash. Only bar soap and liquid soap were observed to be used by participants fr handwashing.

*P-value calculated with Fisher's exact test by household level. No OR was calculated due to the small sample size.

CATI = case-area targeted intervention
